# Supplementary material for: Copy number variation introduced by a massive mobile element facilitates global thermal adaptation in a fungal wheat pathogen
Source: Nat Commun. 2024 Jul 8;15:5728. doi: 10.1038/s41467-024-49913-7 (PMC11231334; doi:10.1038/s41467-024-49913-7)
Supplement: Supplementary file 1 — Supplementary Information [file 41467_2024_49913_MOESM1_ESM.pdf]

Supplementary Figures

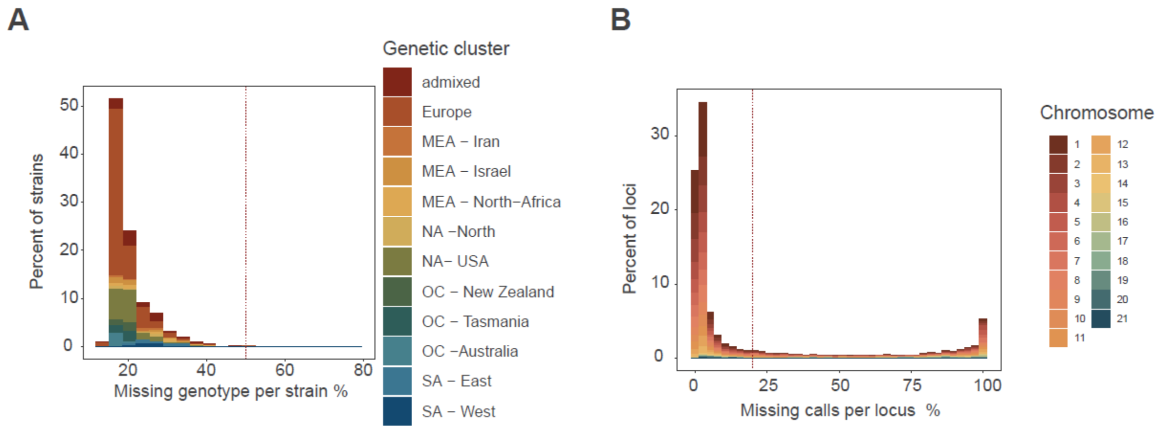

Supplementary Figure 1. Filtering CNV dataset for missing data. A) Distribution of missing genotypes per strain in the CNV call dataset. N = 1104 samples. The red dotted line refers to the 50% threshold. B) Distribution of missing calls per locus (*i.e.* gene) in the CNV call dataset. The red dotted line refers to the 20% threshold.

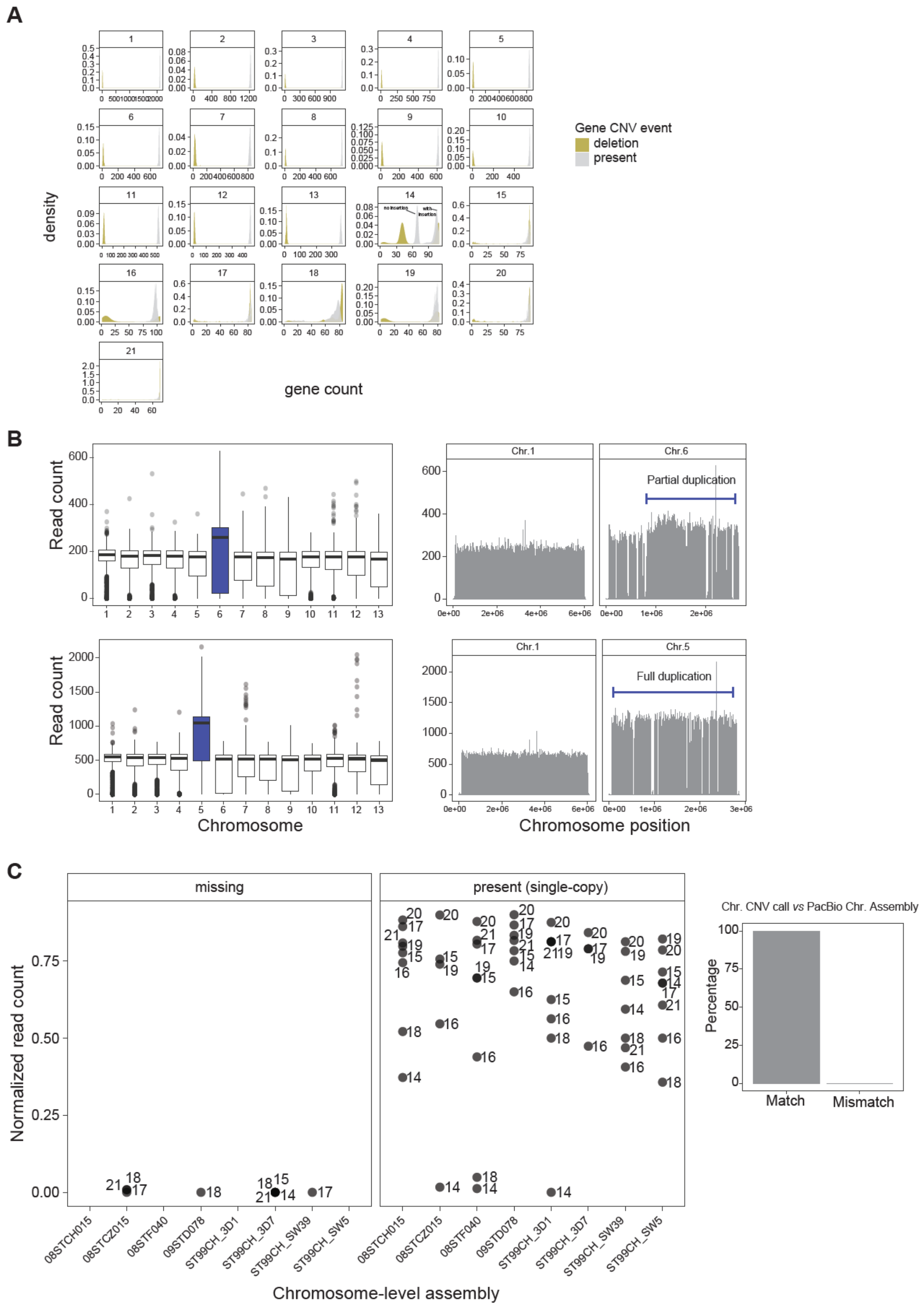

Supplementary Figure 2. Defining chromosome CNV. A) Gene presence/absence density per chromosome in the global genome panel. N = 1104 samples. A cutoff of 60% gene absent calls across the chromosome was applied to label a chromosome as missing. A cutoff of a minimum of 60% gene duplication calls were used to label a

chromosome partially or fully duplicated. Chromosome 14 shows two distinct gene presence peaks due to the large insertion segregating among strains. Colors identify gene CNV events. B) Two representative strains showing partial and full core chromosome duplications. We applied a read coverage threshold of 1.5 times the median core chromosome coverage to define duplications (including partial duplications). Blue color highlights duplicated chromosome. C) Chromosome CNV validation comparing the call method and chromosome-level assemblies from PacBio reads. The left panel y-axis refers to Illumina read coverage and the y-axis refers to the matching PacBio assembly available for the same strain. N = 8 samples. The right panel refers to the number of chromosome matches between CNV calling and assembly.

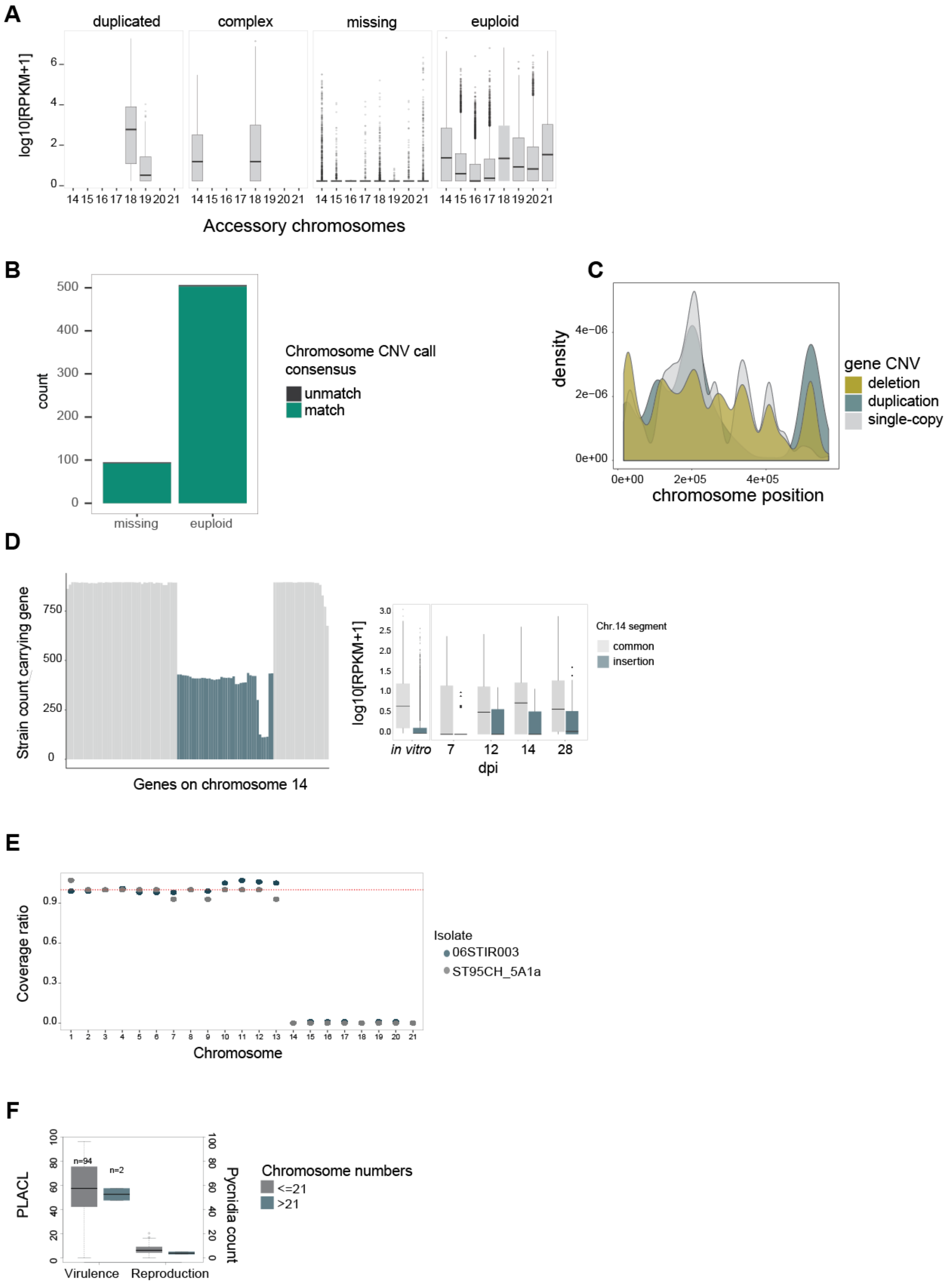
A
duplicated
complex
missing
euploid
log10[RPKM+1]
0
2
4
6
14 15 16 17 18 19 20 21
Accessory chromosomes
B
count
0
100
200
300
400
500
missing
euploid
Chromosome CNV call consensus
unmatch
match
C
density
0e+00
2e−06
4e−06
0e+00
2e+05
4e+05
chromosome position
gene CNV
deletion
duplication
single-copy
D
Strain count carrying gene
0
250
500
750
Genes on chromosome 14
log10[RPKM+1]
0.0
0.5
1.0
1.5
2.0
2.5
3.0
in vitro
7
12
14
28
dpi
Chr.14 segment
common
insertion
E
Coverage ratio
0.0
0.3
0.6
0.9
1 2 3 4 5 6 7 8 9 10 11 12 13 14 15 16 17 18 19 20 21
Chromosome
Isolate
06STIR003
ST95CH_5A1a
F
PLACL
0
20
40
60
80
100
n=94
n=2
Virulence
Reproduction
Pycnidia count
Chromosome numbers
<=21
>21

Supplementary Figure 3. Chromosome CNV variation. A) Transcriptional activity assessment of chromosomes under *in vitro* conditions on a subset of the global panel (n=74 strains) Abraham et al. (2023) to evaluate the robustness of the chromosome CNV calls. Boxes show different types of chromosome CNV calls. B) Number of matches of chromosome CNV calls with loci PCR validation (Croll et al. 2013) (two core and eight accessory chromosome genes tested in 59 strains). The comparison shows matches with the filtered chr. CNV callset. We used euploid and missing chromosome call. We defined a missing chromosome based on the PCR validation when 45% or more of the tested loci of the respective chromosome was absent. C) Distribution of gene CNV events for the chromosome 18 arm showing large CNV polymorphism. D) Chromosome 14 insertion frequencies across populations, as well as gene expression *in vitro* and during wheat infection (Palma-Guerrero, et al. 2016). Dpi refers to days post infection. RNASeq analyses were performed in triplicates. E) Read coverage ratio per chromosome for strains carrying solely core chromosomes. Accessory chromosomes showed no coverage as expected. F) Virulence expressed as Percent Leaf Area Covered by Lesions (PLACL) and reproduction (pycnidia count) on the wheat host of strains carrying variable total chromosome numbers (Singh et al. 2021). The box center line represents the median, and the limits represent the first and third quartiles. Whiskers indicate maximum and minimum values. N = 96 samples.

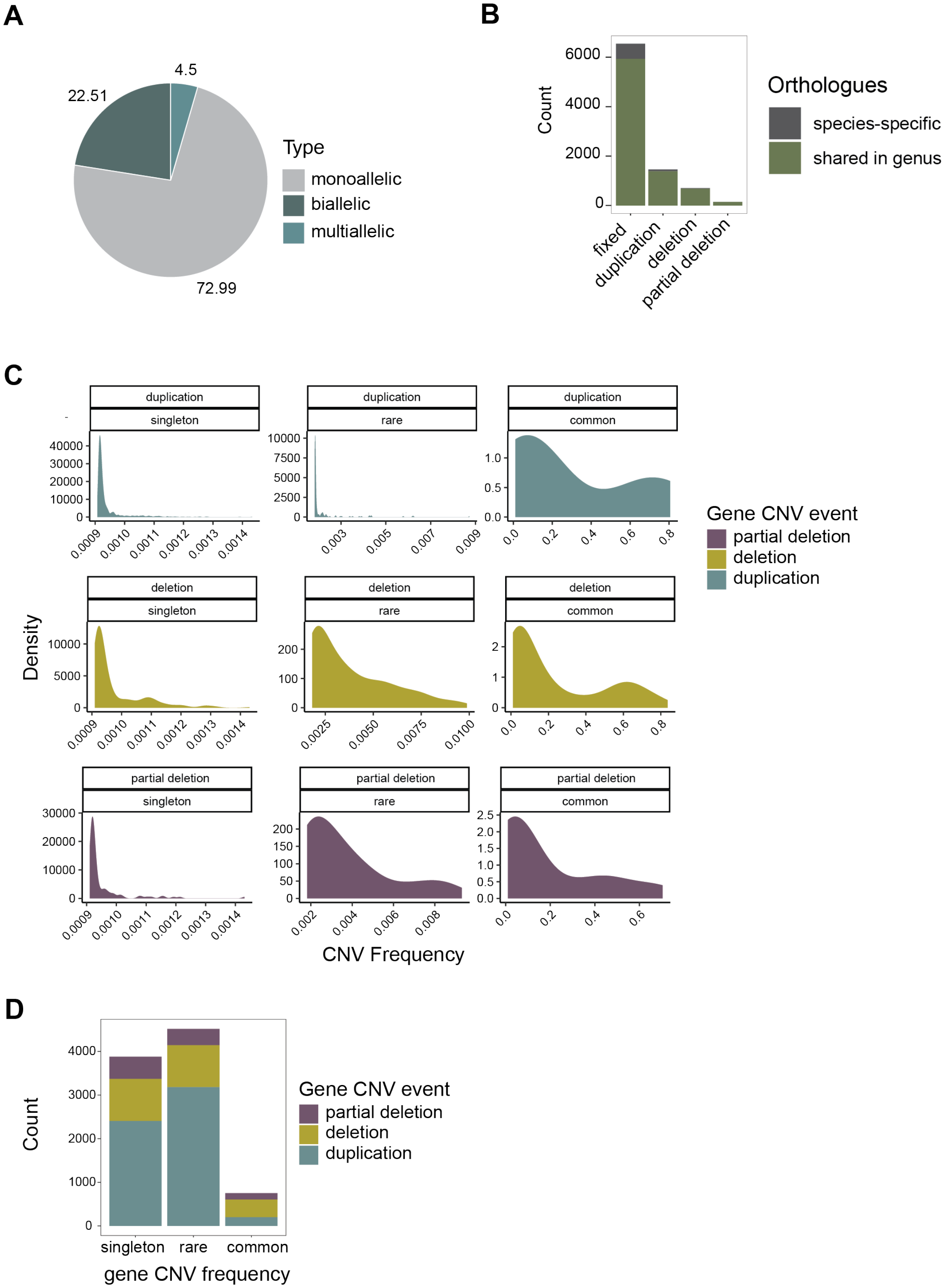

Supplementary Figure 4. CNV frequencies across populations. A) Pie chart showing the overall percentage of alternative alleles (*i.e.* deletion or duplication) in the global genome panel. N = 1104 samples. Multiallelic refers to genes showing multiple types of events. B) Distribution of orthologs shared between sister species and species-specific genes (*i.e.* unique to *Z. tritici*). C) Density plot showing CNV event distribution of each CNV type. D) Gene CNV events for each CNV frequency type in the unfiltered CNV call dataset.

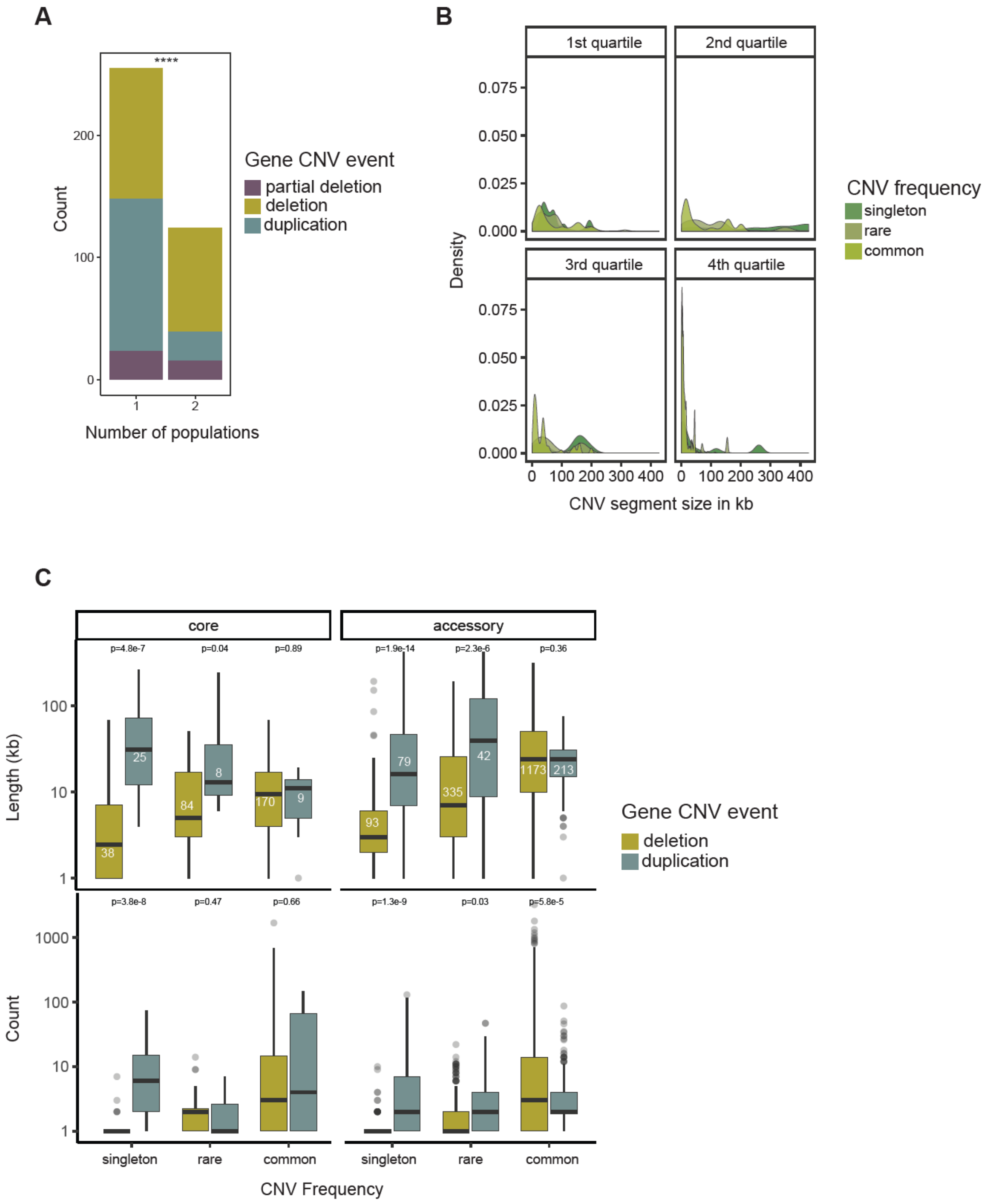

Supplementary Figure 5. CNV features across populations. A) Odds ratio of shared rare frequency gene deletions and duplication in single a population versus population pairs (**** refers to *p value* < 0.0001). B) CNV segment size variation in the global collection shown separately for each quartile of the CNV segment quality score (QA). C) Overall CNV segment size variation and number of genes per segment across CNV frequency and CNV event categories in core and accessory chromosomes. The box center line represents the median, and the limits represent the first and third quartiles. Whiskers indicate maximum and minimum values. Values within boxplots refer to the sample size.

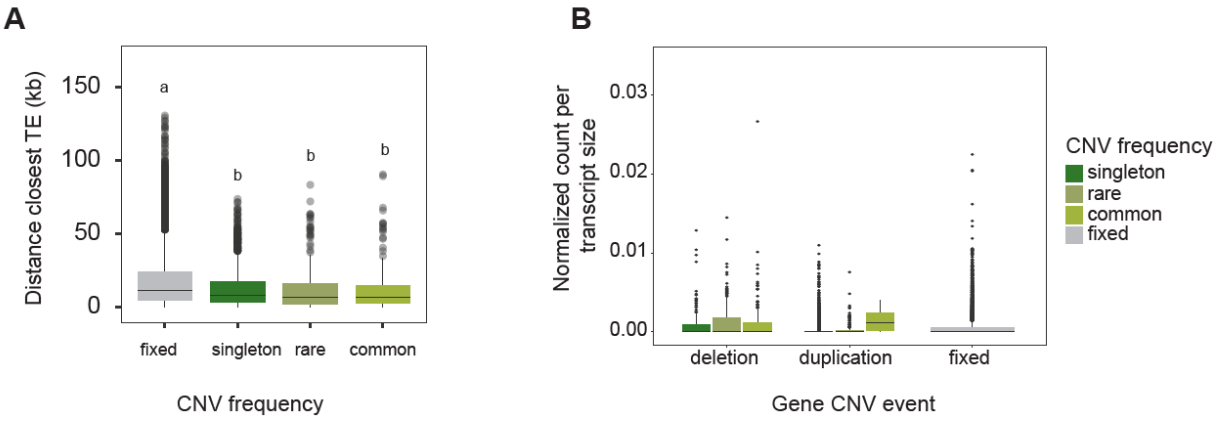

Supplementary Figure 6. A) Distance to the closest TE for each gene CNV frequency category. Letters indicate significant differences (one-way ANOVA followed by Tukey test *p value* < 0.05). B) Predicted protein high-impact SNV variants across gene CNV event categories. n = 8164 loci. Values were normalized by transcript length. In the boxplots the box center line represents the median, and the limits represent the first and third quartiles. Whiskers indicate maximum and minimum values.

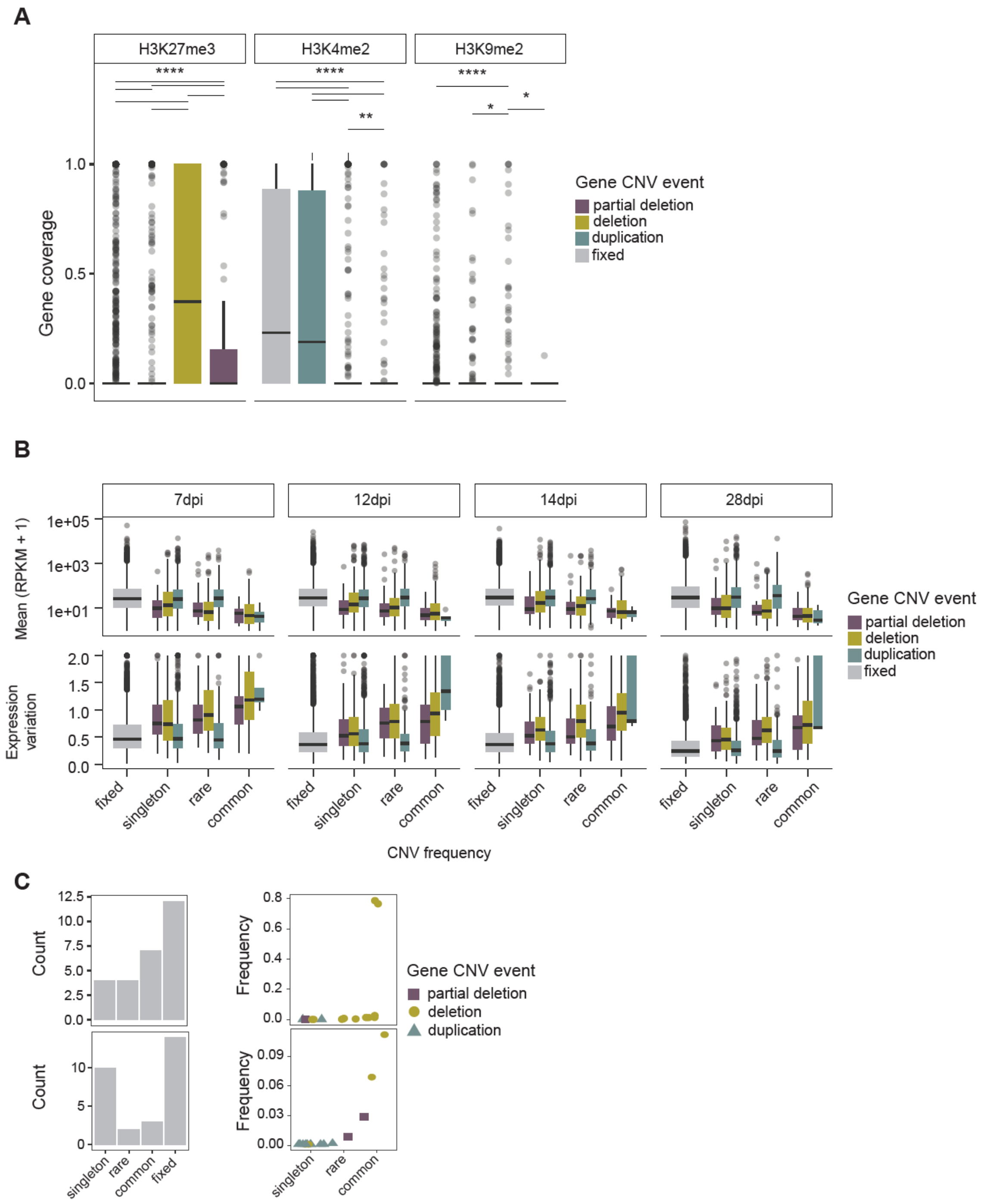

Supplementary Figure 7. A) Gene coverage distribution of histone H3K27me3, H3K4me2 and H3K9me2 methylation marks for gene CNV events. Pairwise Wilcox test. ****, ***, **, * refers to *p values* < 0.0001, <0.0001, <0.01 and < 0.05, respectively. N = 8517 loci. B) Gene expression analysis and expression variation during a host infection cycle (7,12,14 and 28 days after infection) across gene CNV events and frequency categories. RNASeq analyses for each condition was performed in triplicates. C) Gene CNV event profile of the most significantly enriched gene ontology (GO) term for biological process (plot above, secondary metabolic process) and molecular function (plot below, serine endopeptidase activity). The boxplot center line represents the median, and the limits represent the first and third quartiles. Whiskers indicate maximum and minimum values.

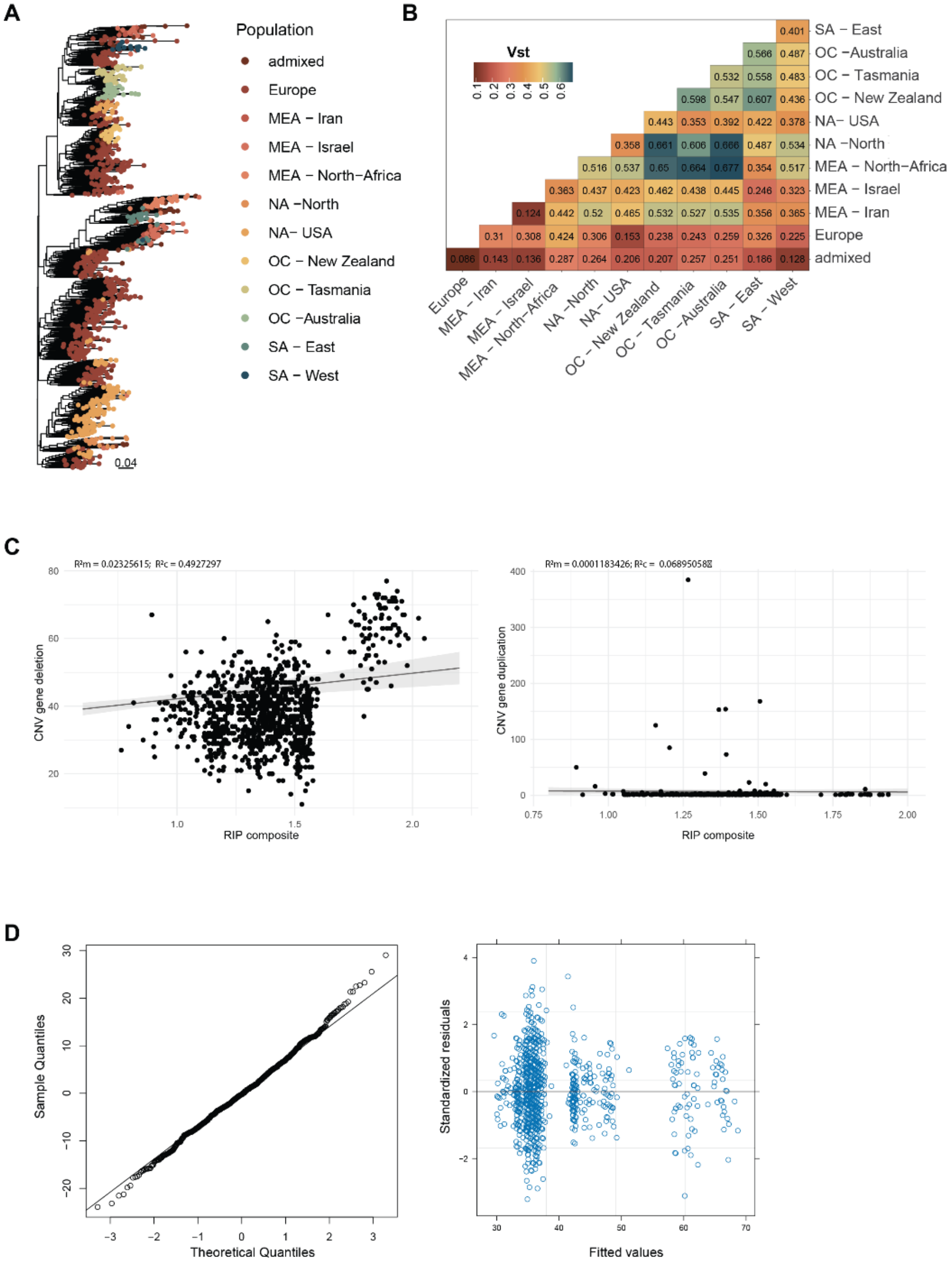

Supplementary Figure 8. A) Neighbor-joining tree based on 136 CNV genes filtered for minor allele frequency >0.05 and based on core chromosome genes. The color scheme identifies genetic clusters inferred by SNV analyses. B) CNV-based population differentiation fixation index V_{ST}. C) Scatter plot of RIP composite index and gene CNV events (left panel: duplications, right panel: deletions). Fitted line of the fixed effect of the predictor variable (RIP composite index) on the response variable (gene CNVs) accounting for the random effect variables (RIP composite | Genetic cluster) based on a linear mixed model. R^2c and R^2m refer to the conditional and marginal coefficient of determination for the generalized mixed-effect models, respectively. (D) Quantile-Quantile (QQ) plot of residuals and residual plot of the linear mixed model, indicating the distribution of residuals (vertical axis) against the fitted values (horizontal axis) for the gene deletion model.

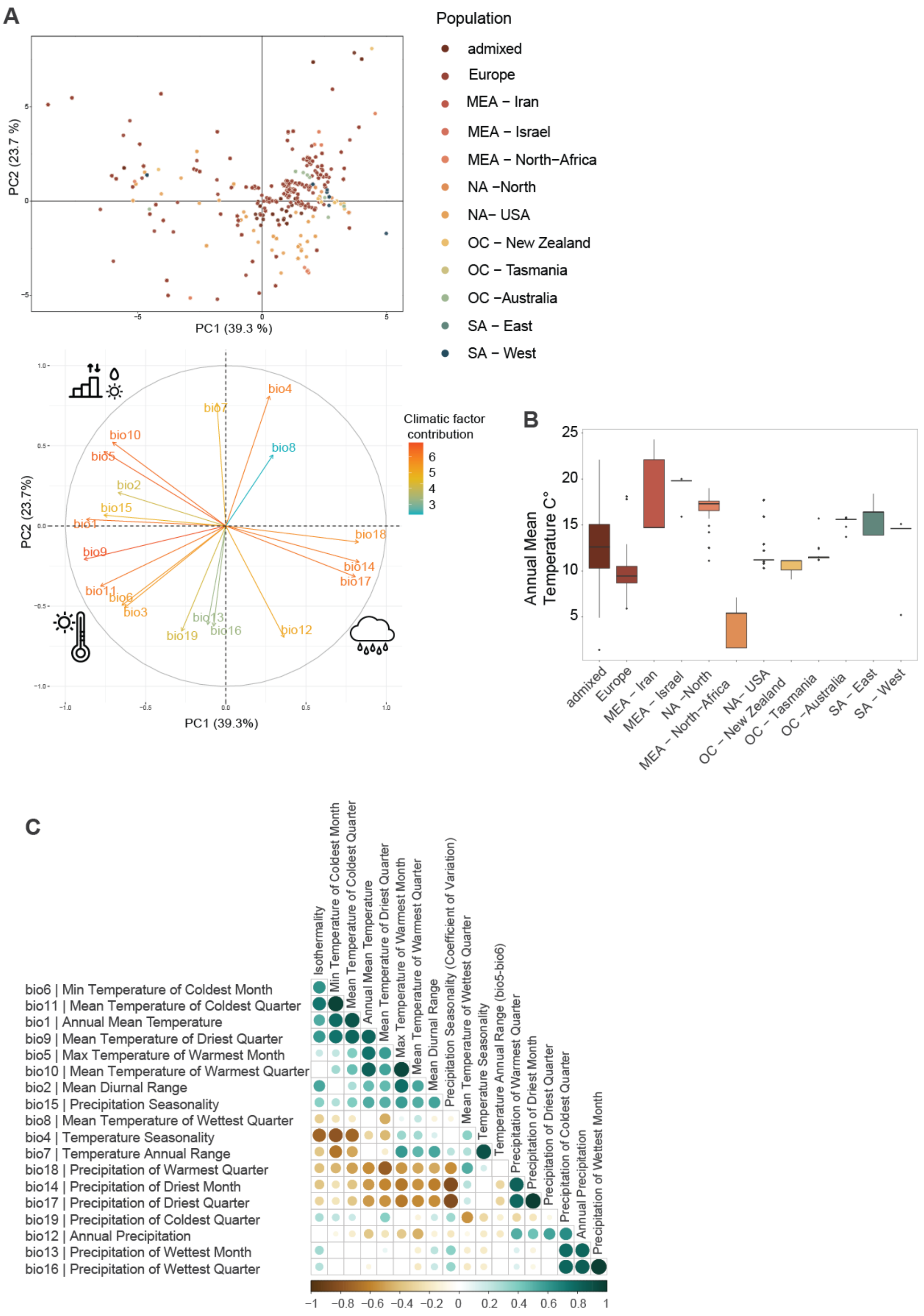

Supplementary Figure 9. Climactic factors available to characterize sampling locations. A) Plot of the first and second principal component based on 19 climatic factors assessed for the geographic location of each sampling location. The color scheme identifies genetic clusters inferred by SNV analyses. The plot below refers to climatic variable contributions to the first and second principal component. Lower-right, lower-left and upper-left areas of the plot represent overall precipitation, temperature and climate range variation variables, respectively. B) Annual mean temperature (bio1) variation between genetic populations. The boxplot center line represents the median, and the limits represent the first and third quartiles. Whiskers indicate maximum and minimum values. N = 1104 samples. C) Correlation plot between the 19 climatic factors used for genotype-environment association (*p value* <0.0001).

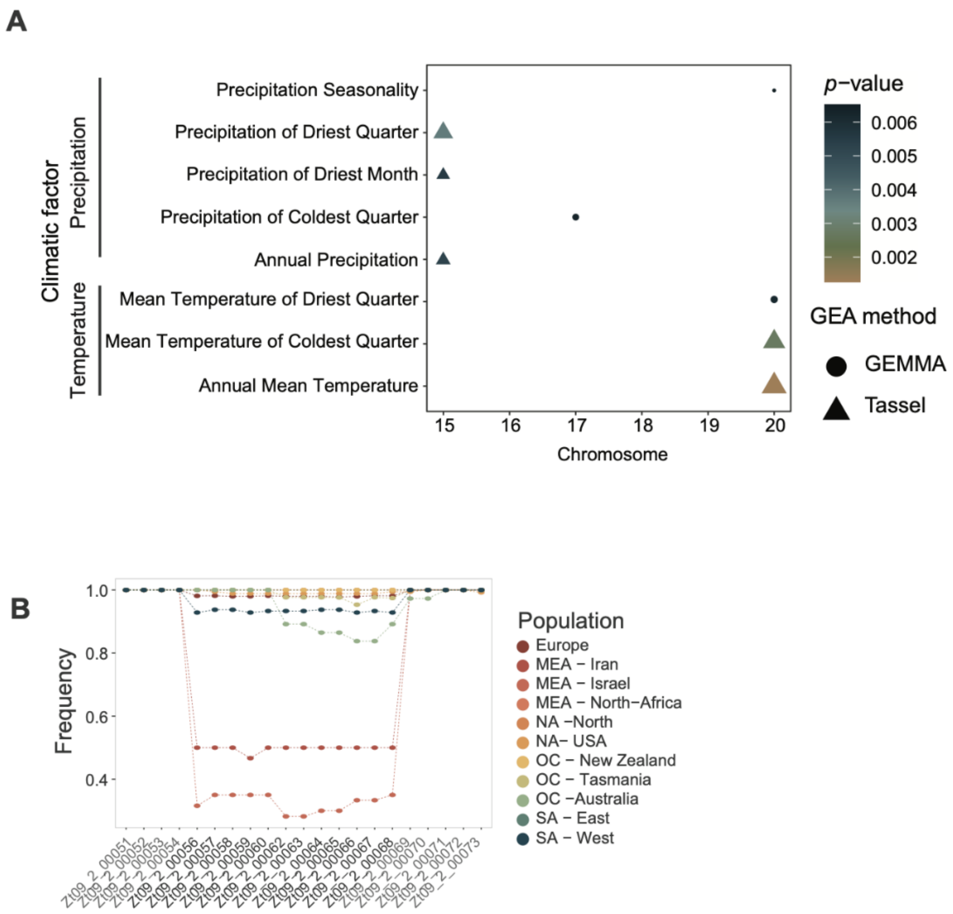

Supplementary Figure 10. A) Significant associations from genome-environment association (GEA) analyses (Bonferroni alpha = 0.05) based on chromosome presence/absence variation for 19 climatic factors assessed for the geographic location of each sampling location using two mixed model methods (implemented in Tassel and GEMMA, respectively). Color and symbol size refer to the *p value* of the associations. Duplicated chromosomes were removed from the analyses. B) BGC19 frequency across the genetic clusters. Genes in grey refer to the flanking regions.

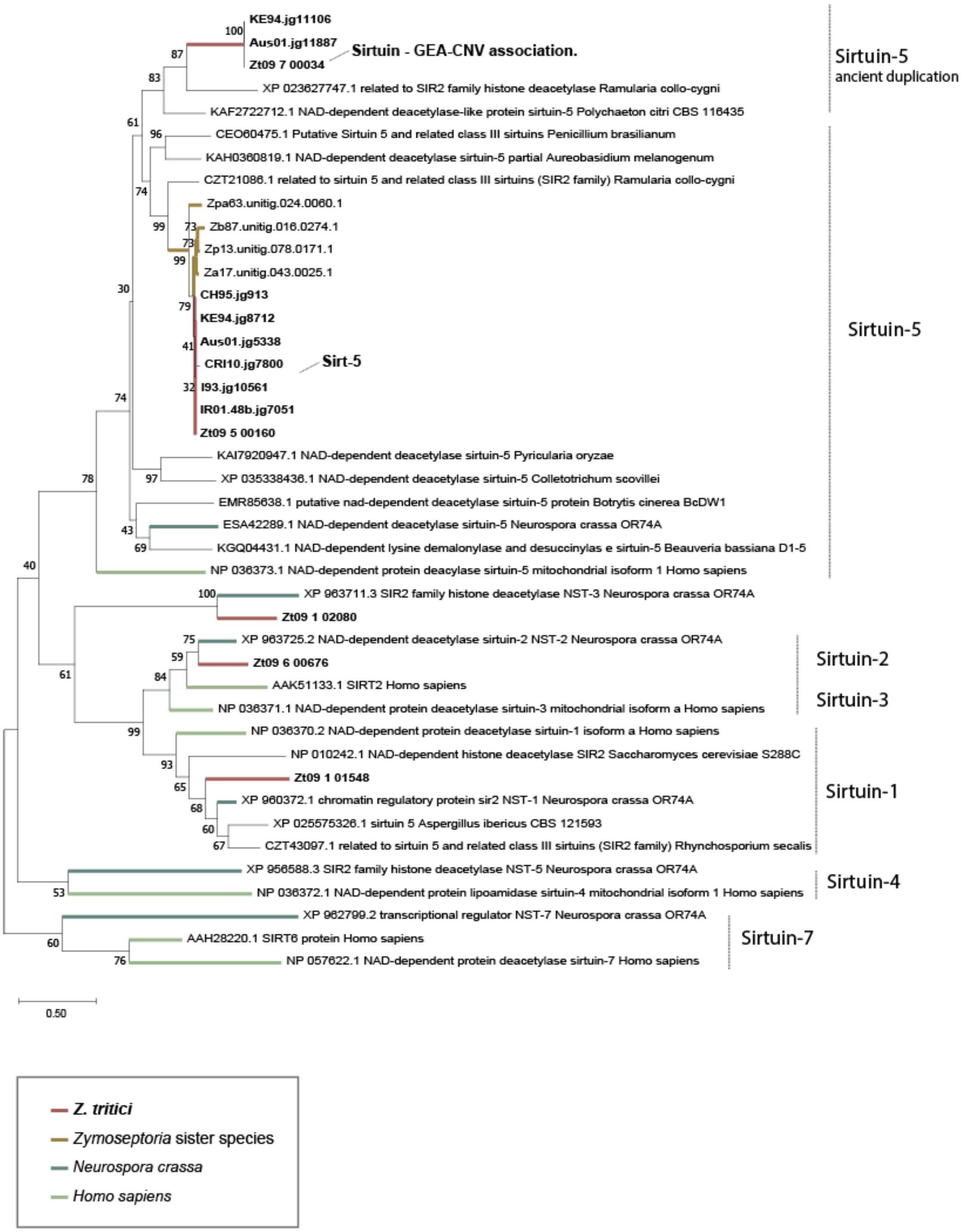

Supplementary Figure 11. Evolutionary history of the *Sirtuin* gene family. The tree topology and *Sirtuin-5* paralogues in *Z. tritici* and *Ramularia collo-cygni* support an ancient duplication of *Sir5* in the Dothideomycetes. Unrooted phylogenetic tree for *Sirtuin* orthologs based on maximum likelihood with 1000 bootstrap replicates. The colored lines refer to individual species. *Sirtuin* clade names on the right of the tree are based on the *Homo sapiens* naming scheme for reference. Sirtuin – GEA-CNV refers to the identified association between CNVs and climatic factors.

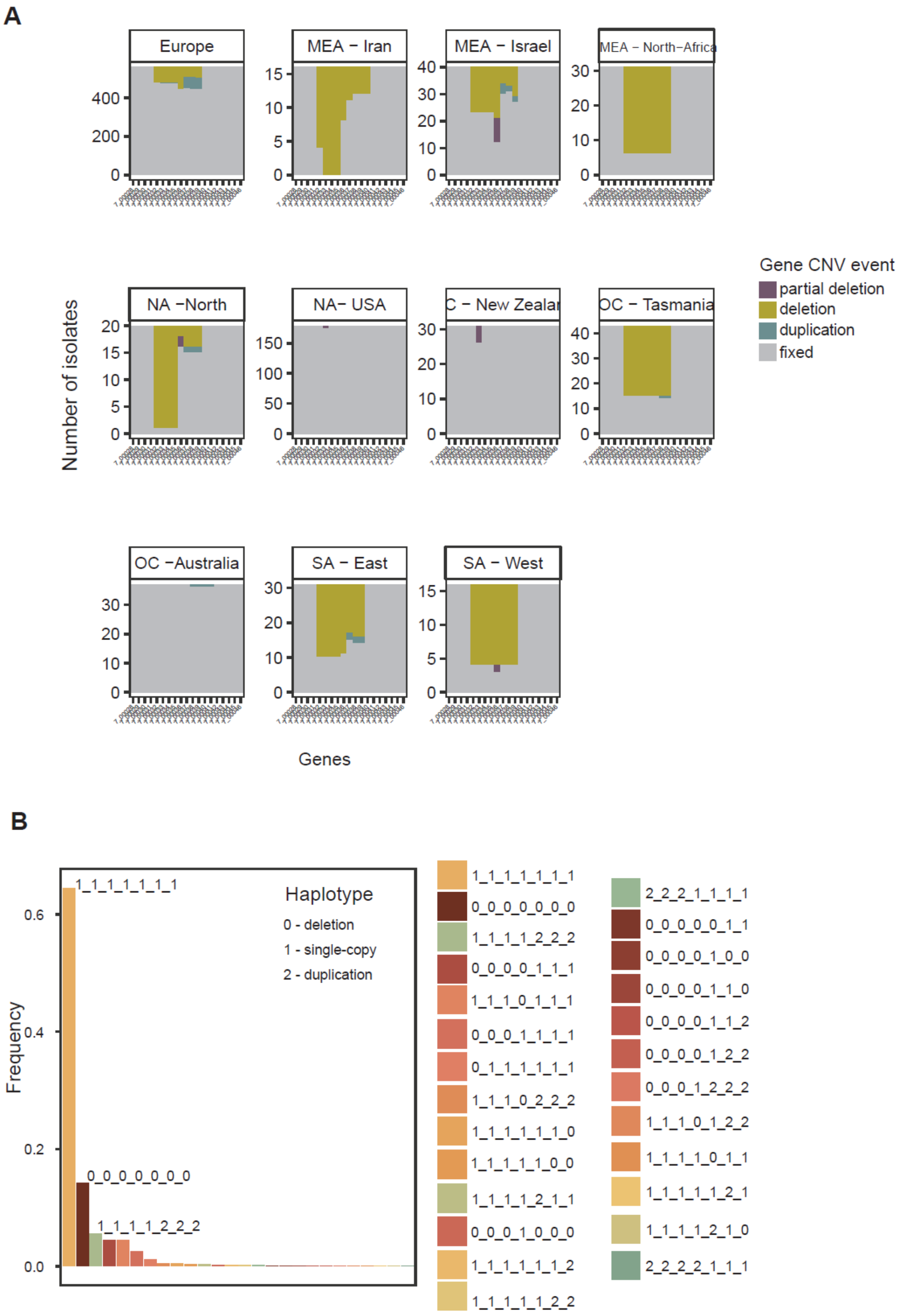

Supplementary Figure 12. A) Gene CNV frequencies in the *Starship* region across isolates from different genetic clusters. N= 1104 samples. B) *Starship* haplotype frequency based on the unfiltered gene CNV dataset. Haplotypes were ordered by decreasing frequency in the global genome panel. Haplotype numbers refer to deletion, single-copy or duplication of the gene. Gene order was based on the IPO323 reference genome.

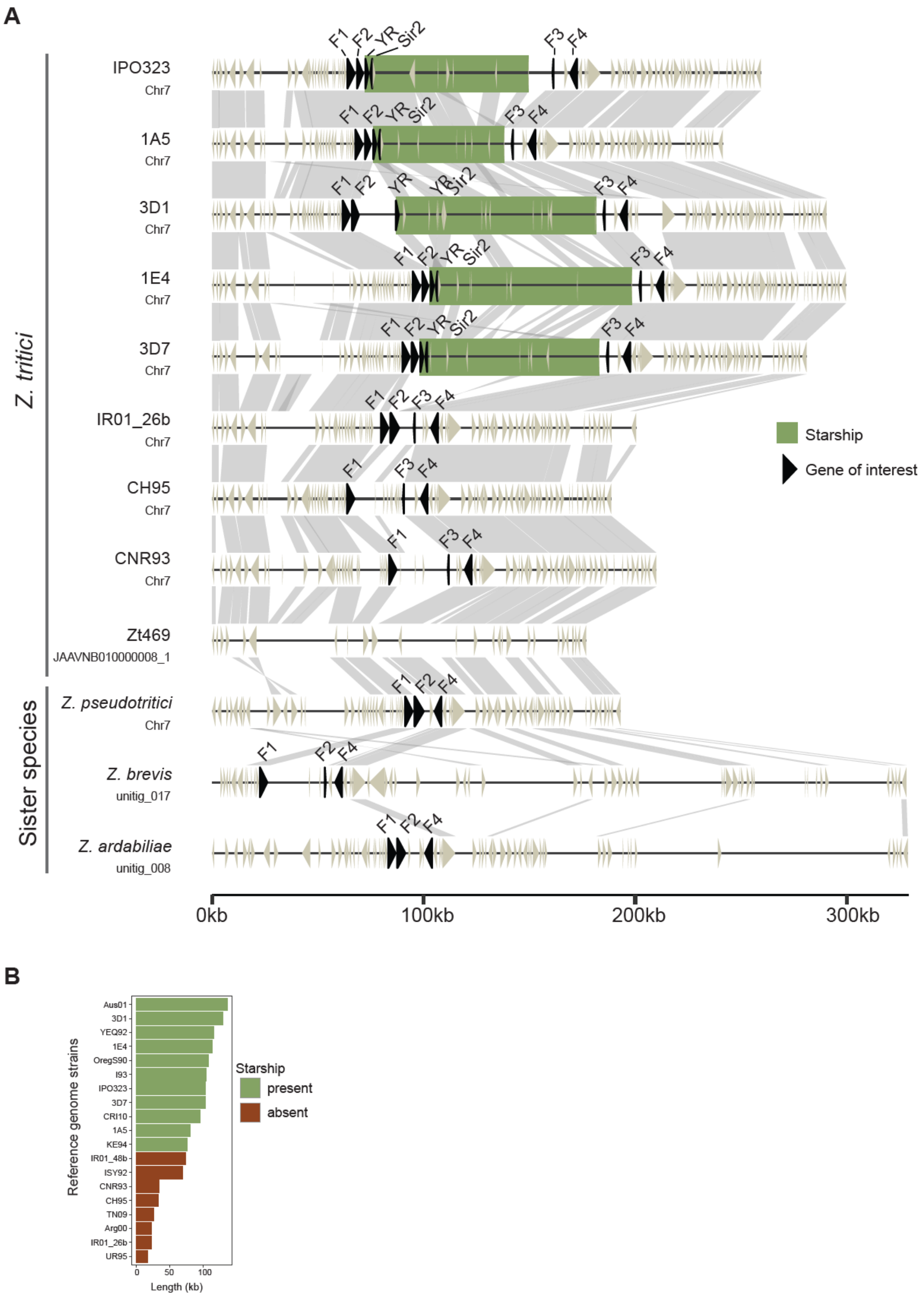

Supplementary Figure 13. A) Genome level synteny plot of the *Starship* region between chromosome-level assemblies of *Z. tritici* and sister species strains. Only alignments ≥1000bp and 90% identity are shown. Predicted genes are displayed as arrows and genes of interest are filled in black, including four genes belonging to ortholog groups that have conserved positions flanking the element. YR: tyrosine recombinase (Zt_7_00033); F1: Zt09_7_00031; F2: Zt09_7_00032; F3: Zt09_7_00040; F4: Zt09_7_00042. The green bar identifies the *Starship*. B) Size between flanking region (Zt09_7_00031 up to Zt09_7_00041) of the *Starship* region. Colors refers to presence or absence of the mobile element.

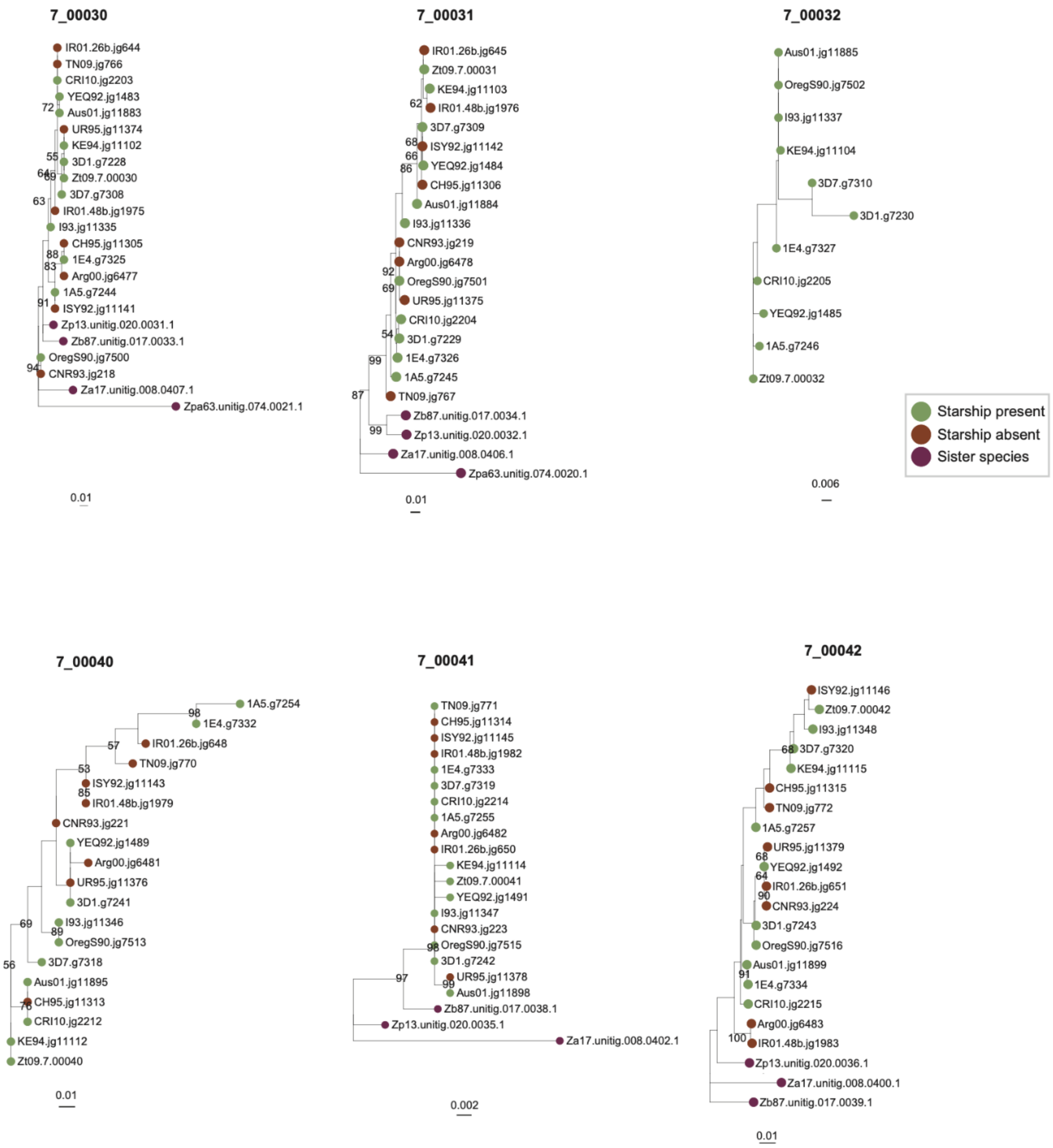

Supplementary Figure 14. Phylogenetic trees of the chromosome-level assemblies and sister species protein sequence orthologues flanking the *Starship* region. Trees were constructed using maximum likelihood with a 1000 bootstrap replicates. Color circles identify the presence or absence of the *Starship* across genomes.

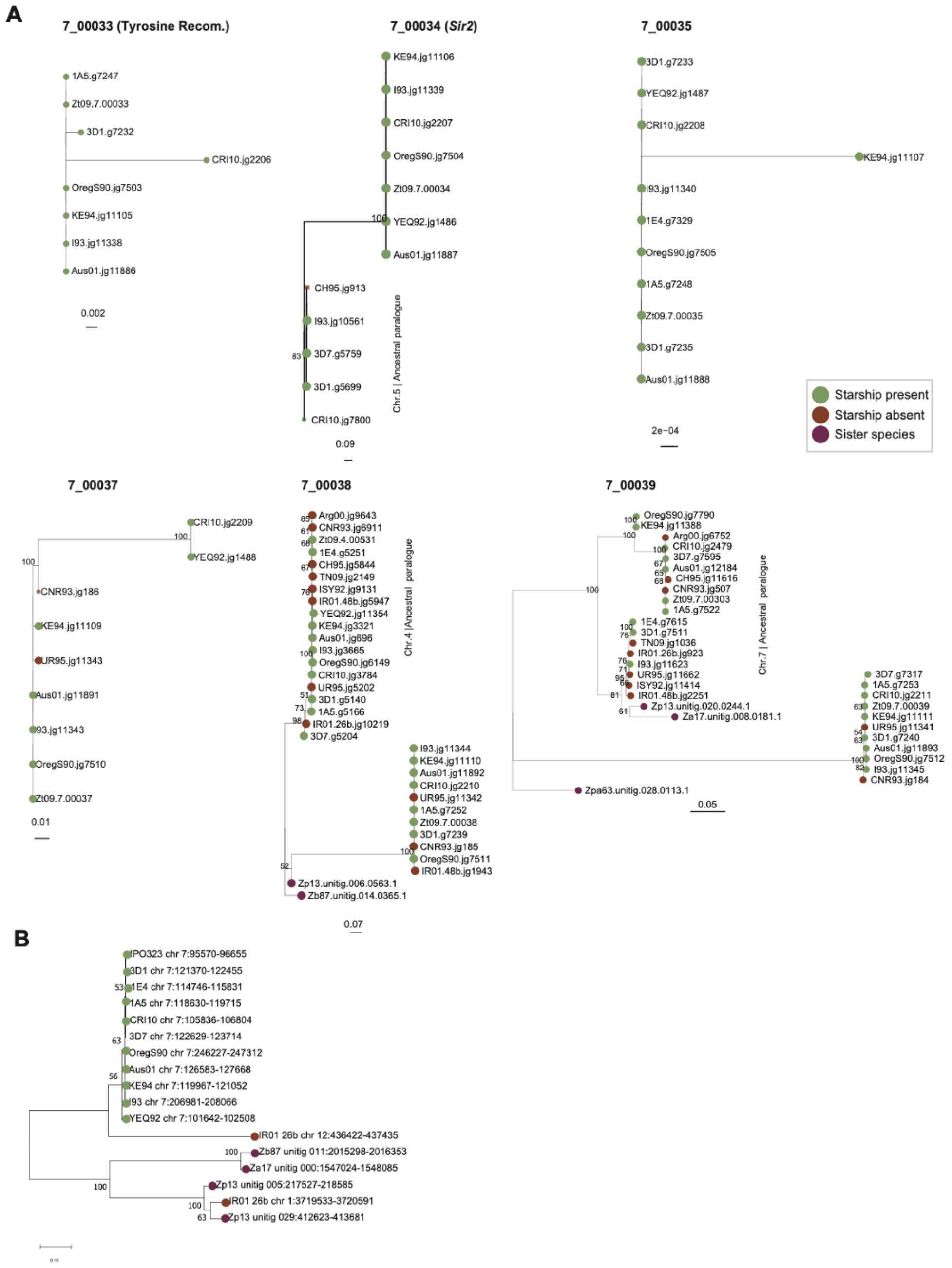

Supplementary Figure 15. A) Phylogenetic trees of the chromosome-level assemblies of *Z. tritici* and sister species protein sequence orthologues within the *Starship* region. Trees were constructed using maximum likelihood with 1000 bootstrap replicates. Color circles refer to presence or absence of the *Starship* across genomes. Paralogues in the tree are highlighted. Names identify strains carrying an orthologue. B) Unrooted phylogenetic tree of the tyrosine recombinase gene based on tblastn analysis and built with maximum likelihood and 1000 bootstrap replicates. Names identify chromosome-level assembly strains and the loci coordinates.

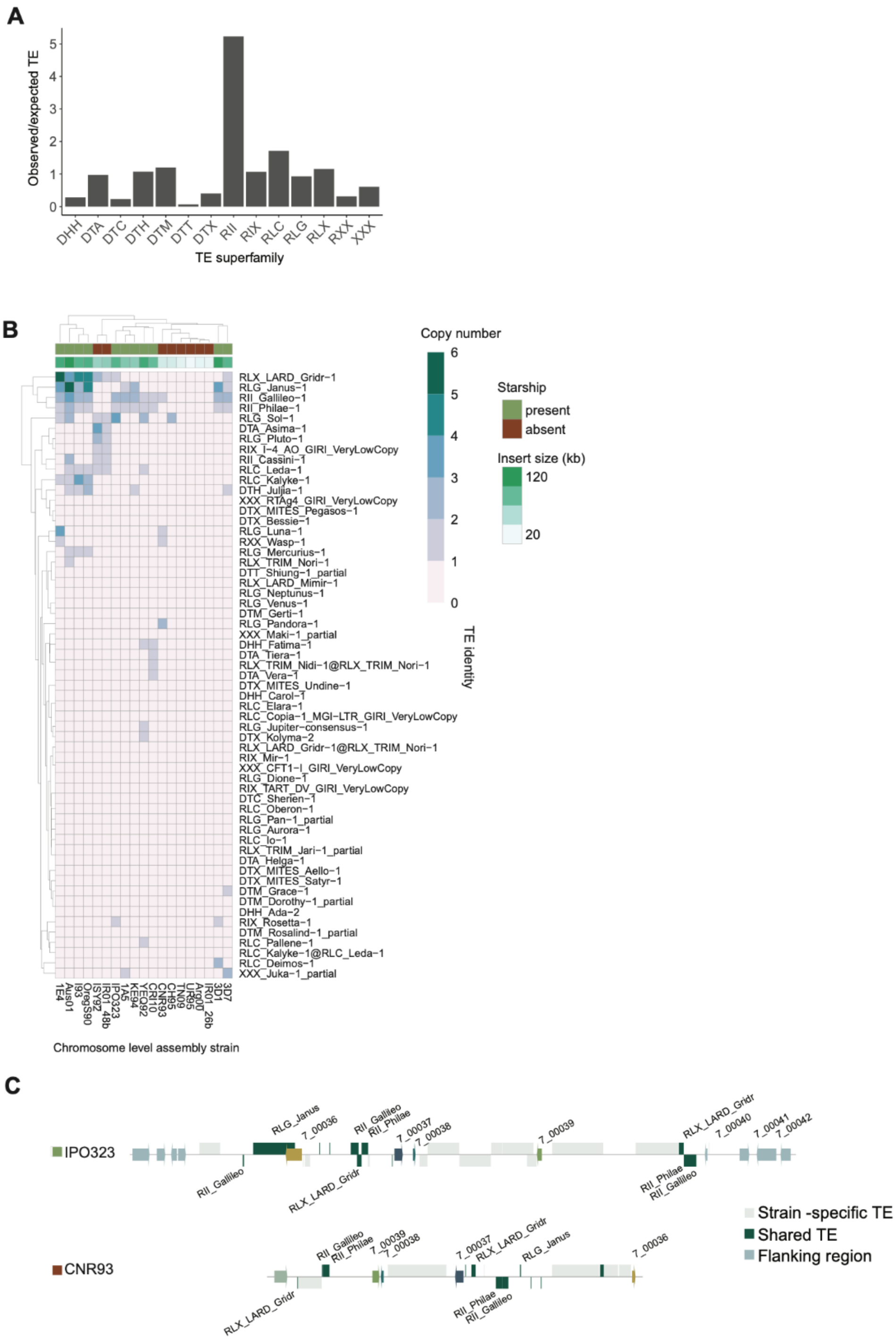

Supplementary Figure 16. A) Barplot showing the overall ratio between the *Starship* region compared to genome-wide transposable element content (core chromosomes) in fully assembled genomes. B) Heatmap of transposable elements within the *Starship* of each chromosome-level assembly. Transposable element annotation was retrieved from Badet et al. 2020. C) Shared TEs between the *Starship* in the IPO323 reference genome and the genomic context in the CNR93 chromosome-level assembly indicating a transposition event of the *Starship* region. Arrows represent genes. Colors identify groups of orthologs. Blocks represent TEs. @ refers to nested TEs.

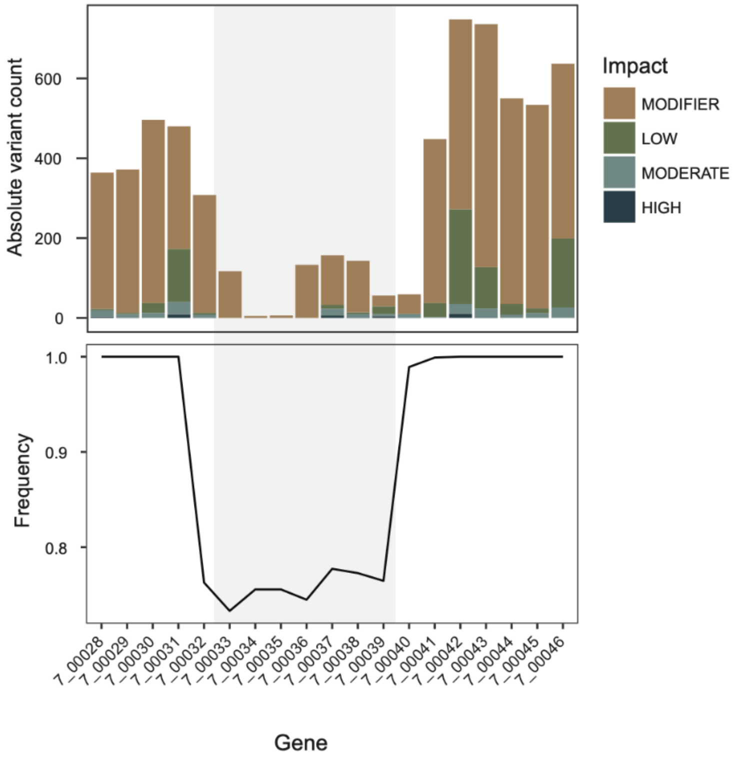

Supplementary Figure 17. Protein variant impact counts expressed as the number of individual mutations and gene presence frequencies in the *Starship* region assessed for the global genome panel (n=1104) suggesting that the genes 7_00034 and 7_00035 are under strong purifying selection. Grey shading identifies *Starship* cargo genes.
