# Supplementary material for: Copy number variation introduced by a massive mobile element facilitates global thermal adaptation in a fungal wheat pathogen
Source: Nat Commun. 2024 Jul 8;15:5728. doi: 10.1038/s41467-024-49913-7 (PMC11231334; doi:10.1038/s41467-024-49913-7)
Supplement: Supplementary file 3 — Description of Additional Supplementary Files [file 41467_2024_49913_MOESM3_ESM.pdf]

Description of Additional Supplementary Information

Supplementary Data 1: Information about sample origin, sequence quality and accession numbers.

Supplementary Data 2: Sample list of independently sequenced sets of identical strains (i.e. independent library and sequencing used to assess CNV calling quality

Supplementary Data 3: Output of the long-read and whole-genome alignment-based structural variant analyses (SyRI method; retrieved from Badet 2021).

Supplementary Data 4. High quality copy number variation (CNV) call of the global panel of *Zymoseptoria tritici* strains. chr_SV refers to chromosome CNV. CNQ refers to the CNV call quality.

Supplementary Data 5: Contiguous CNV calls binned to CNV segment.

Supplementary Data 6: Enrichment of gene ontology terms in CNV genes

Supplementary Data 7: *Vst* values and functional annotation for CNV genes

Supplementary Data 8: Climatic variables retrieved from WorldClim database used for CNV-GEA analysis.

Supplementary Data 9: Significant GEA s associations between chromosome presence/absence and climatic variables after Bonferroni adjustment.

Supplementary Data 10: Phenotypic traits of reproduction and virulence of Z. tritici isolates grown on 12 distinct wheat cultivars. Data retrieved from Dutta et al. 2021.

Supplementary Data 11: GEA-gene CNV associations after Bonferroni adjustment using two methods (Gemma and Tassel).
